# Adverse Birth Outcomes and Maternal Morbidity Among Afro-Latinas and Their Infants: A Systematic Literature Review

**DOI:** 10.1007/s40615-024-02107-9

**Published:** 2024-08-14

**Authors:** Alexa Parra, Vanessa Morales, Cynthia N. Lebron, JoNell Potter, Yue Pan, Hudson P. Santos

**Affiliations:** 1 School of Nursing and Health Studies, University of Miami, 5030 Brunson Dr, Coral Gables, FL 33146, USA; 2 Miller School of Medicine, Department of Public Health, University of Miami, 1120 NW 14Th St, Miami, FL 33136, USA

**Keywords:** Maternal and child health, Afro-Latina, Black Latina, Black Hispanic, Maternal morbidities, Infant birth outcomes

## Abstract

**Objectives:**

To evaluate and synthesize research findings on adverse birth outcomes and maternal morbidity among Afro-Latinas and their infants.

**Methods:**

A systematic review was conducted within PubMed, Web of Science, and SCOPUS databases. Four thousand five hundred twenty-six published peer-reviewed articles from 1970 to 2023 that reported outcomes related to maternal morbidity and/or birth outcomes were screened. After screening, we assessed 22 for eligibility, and ultimately, seven studies were included for data extraction and analysis.

**Results:**

Although limited, the existing studies revealed disparities in abnormal birth weight (LBW & SGA) and higher preterm birth prevalence among Afro-Latinas compared to other racial and ethnic peers. These disparities are also prevalent among U.S.-born Afro-Latinas compared to foreign-born Afro-Latinas.

**Conclusions:**

By critically examining the current empirical evidence, we can gain a deeper understanding of how intersectionality impacts perinatal health outcomes among Afro-Latinas. Understanding the root causes of these outcomes through increased research is critical to preventing and reducing poor maternal and child health among Afro-Latinas, particularly those who are U.S.-born.

## Introduction

Addressing maternal and child health (MCH) disparities among racially Black women in the United States (U.S.) cannot be stressed enough. Maternal morbidity and mortality have significantly increased in the past 20 years, with the largest rise seen in women who identify as Black. Although infant mortality has not experienced as substantial an increase, there is still a significant racial inequity. In light of the ongoing discussions around racial inequities in birth outcomes, it is essential to gain a more nuanced understanding of the variations within the population [1–3]. Traditional categorizations of race and ethnicity may inadvertently mask disparities in birth outcomes [3].

Despite remarkable advancements in research on perinatal Latina women and their infants, the current body of research needs to account for the comprehensive representation of the heterogeneity within the Latin community, particularly among Afro-Latinas [1, 4]. Afro-Latinas refers to individuals of African descent from Latin America and the Caribbean [5] who identify as Black and ethnically Latina (and/or Hispanic) [4] or are racially perceived as Black by others [1, 6]. Although approximately 17% of Afro-Latino/a’s (in the U.S.) do not identify as racially Black, the majority identifies as racially Black and ethnically Latino and/or Hispanic [7]. Terms such as Afro-Latinas, Black Latinas, Afro-Hispanics, and Black Hispanics are used interchangeably to describe individuals who self-identify as Black or of African descent *and* Latina and/or Hispanic [1, 8, 9]. Afro-Latinas will be used hereafter when referring to studies with participants identifying as Black and Latina/Hispanic.

Historically, in the U.S., Black identity is synonymously used to describe African American people, which may make it challenging for Latinos of African descent to self-select into the Black racial category. However, over the last two decades, there has been a large increase in self-identified Afro-Latino/as [8, 10], with younger Latino generations who are brought up in the U.S. are more likely to use established racial categories, while older generations may not [10]. Notably, there was a significant increase in the number of Hispanics identifying as “two or more races” from 6% in 2010 to 32.7% in 2020, with approximately 24% considering themselves Afro-Latino [8, 10].

Little is known about perinatal outcomes for Afro-Latina women and their infants, who may face unique disparities in health outcomes due to the intersection of social stressors related to multiple minority identities [1, 4]. The burden of health disparities may be more pervasive among Afro-Latinas, as they may experience distinctive stressors related to societal disadvantages, including race and skin color, in ways distinct from other Latinos (e.g., white Latinos) [11, 12]. The lived experiences of Afro-Latinos share similarities with non-Latino Black people, such as increased risk of discrimination, health-related risks, and possible poor pregnancy outcomes due to their increased exposure to chronic stressors across the life course [2, 4, 13, 14]. Current literature on Latinas, traditionally focused on non-Black Latinas, implies that they are not disproportionately affected by maternal and birth disparities compared to their non-Latina Black counterparts [4]. Notably, the extent to which the confluence of being Black and Latina is a risk factor for these maternal morbidities and infant birth outcomes is poorly understood [5]. Determining the prevalence of these adverse perinatal and infant outcomes among Afro-Latinas is essential to optimizing perinatal outcomes [6, 15–17].

This systematic review evaluates the literature available on adverse birth outcomes and maternal morbidity among Afro-Latinas and their infants. By critically appraising these outcomes, we can better understand the intersection of race and ethnicity and determine this convergence’s relative impact [2].

## Methods

### Defining Afro-Latinas

The term “Afro-Latina” is a multifaceted identifier commonly used to describe individuals of African descent who identify as Latina and/or Hispanic [18]. However, Afro-Latina is not a universally standardized term in data collection, census, or research, making it challenging to capture a comprehensive and accurate representation of this demographic group [18]. For this study, our team used the term “Afro-Latinas” as an inclusive descriptor to encompass the various ways this demographic group was identified in the reviewed articles, which included (not limited to) the following designations: Black Latina [11, 19], Afro-Caribbean [20], South and Central American Black, and Puerto Rican Black [21], and Hispanic Black [5].

### Eligibility Criteria

A systematic review was conducted to collect and synthesize research findings on maternal morbidity and adverse birth outcomes among Afro-Latinas and their infants. Maternal morbidity was determined using the World Health Organization (WHO) definition as “any health condition attributed to and/or aggravated by pregnancy and childbirth that has a negative impact on the woman’s wellbeing” [22]. We included empirical and quantitative articles and other literature (e.g., published studies and reports) detailing original research. Studies were included if they: (1) included Afro-Latina women or participants identified as Black and Latina/Hispanic (race and ethnicity categories); (2) quantitatively examined the prevalence of maternal morbidity and/or adverse birth outcomes among this population. To ensure comprehensive coverage of maternal morbidity and birth outcomes, we did not exclude articles that focused on perinatal women with severe co-morbidities (e.g., HIV, twin gestation). Further, because this is a systematic review on Afro-Latinas (Black women), we included a range of factors that have historically been studied in other groups of racially Black perinatal women, such as gestational diabetes, hypertensive disorders of pregnancy, infant birth weight, and preterm birth. The Preferred Reporting Items for Systematic Reviews (PRISMA) recommendations were used as a guide for the methods of this review.

### Information Sources and Search Strategy

We conducted comprehensive literature searches within PubMed, Web of Science, and SCOPUS for studies that examined maternal morbidity and adverse birth outcomes among Afro-Latinas. The study protocol was created using the Methodological Expectations of Cochrane Intervention Reviews (MECIR) (C1–C23) and registered with Prospero [23] (CRD42023404206). Our search strategy was limited to (1) peer-reviewed journal articles, (2) published within the years 1970–2023 (1970 is when the United States census started to include Latino/Hispanic origins), (3) location was not restricted (meaning studies that were conducted outside of the United States were eligible), (4) language was not restricted but needed to be translated to English for review. We applied search terms (this includes variants of word forms) systematically across all databases to capture: (1) the population of interest (Afro-Latina); (2) the exposure period of interest (the perinatal period starting at 22 completed weeks gestation and lasting through seven days after birth [24]); and (3) specific outcomes of interest (e.g., gestational diabetes, hypertension disorders of pregnancy, preterm birth).

We searched the following terms: population of interest (“Afro-Latina* OR Black-Hispanic *OR Black Latina* OR Afro-Hispanic*”), exposure of interest (“Pregnancy* OR perinatal *), and outcomes of interest (“Morbidity* OR Pregnancy Complications* OR Diabetes, Gestational* OR Hypertension, Pregnancy-Induced* OR Premature Birth*). Our search was conducted between March and July of 2023. Bibliographies from selected articles were appraised to identify additional relevant literature cited.

### Study Selection Process

**Fig. 1:**
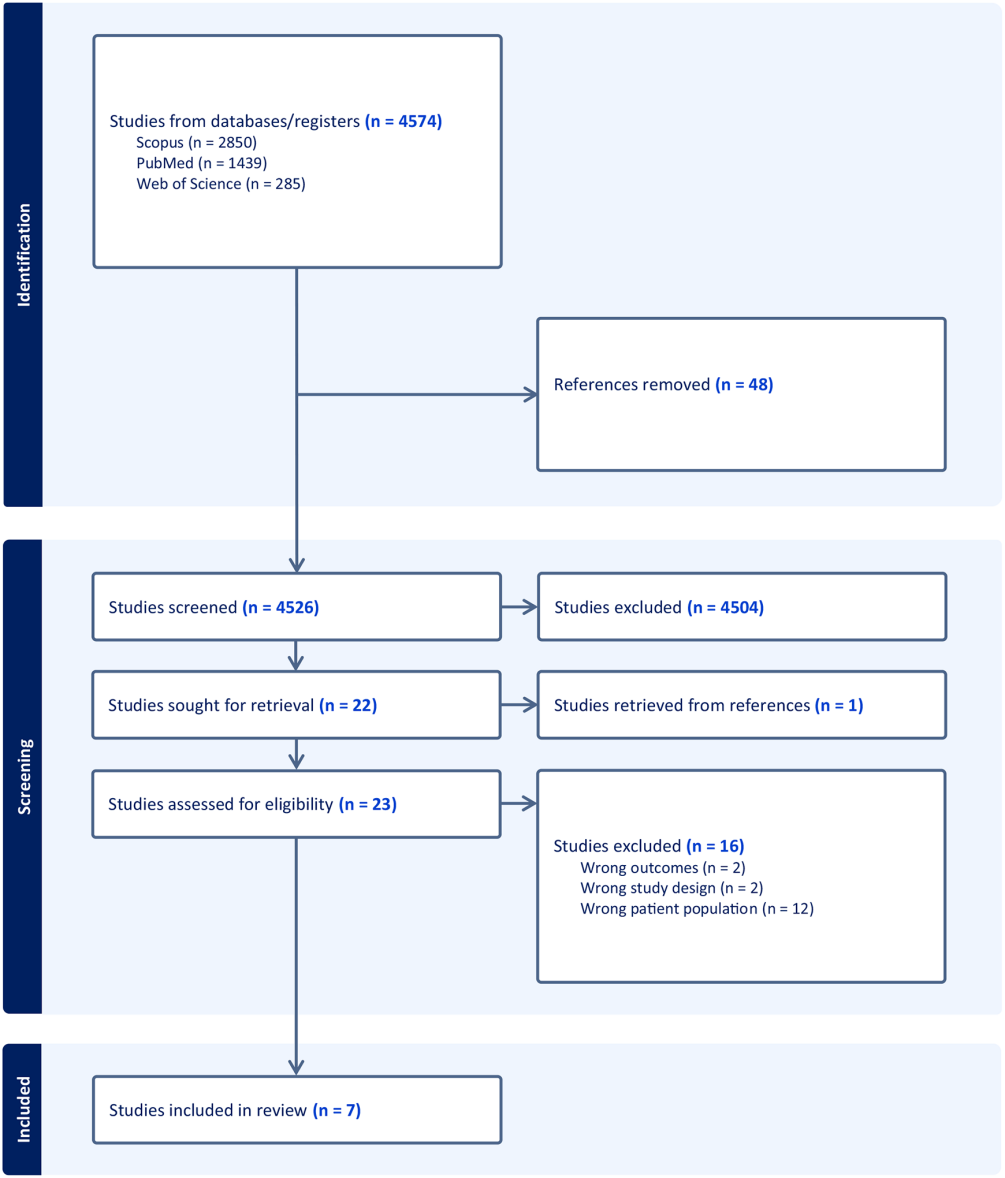
Covidence generated PRISMA flowchart

Our search originated 4579 articles, and all were imported into Covidence^™^, a screening and data extraction tool, and were screened for duplication (Fig. 1, Prisma flowchart). After duplicates were excluded (*N* = 4526), titles and abstracts were assessed by two independent (AP and VM) reviewers to identify those that appropriately addressed the review’s objectives and met the inclusion criteria; a third reviewer (CL) addressed conflicts between the two independent reviewers. In the title and abstract screening, 4504 articles were excluded. For the remaining 22 articles, we reviewed the full text to determine final inclusion. Six articles met inclusion criteria, and one article from the references was identified for extraction and met inclusion criteria. As the final sample, we included seven articles in this systematic review. All conflicts were resolved through consultation between reviewers until a consensus was reached.

### Extraction and Synthesis

A data extraction template was constructed in Covidence^™^, which included title, author, year of publication, country in which the study was conducted, aims, study design, participants (number, description), inclusion/exclusion criteria, maternal morbidity, and adverse birth outcome variables, and key findings (see Table 1). The synthesis phase of our systematic review consisted of the Covidence^™^-generated PRISMA flowchart describing the search and screening of articles, followed by a quantitative analysis of extracted study characteristics.

## Results

The final data analysis included findings from 7 cross-sectional studies published from 2002 to 2022. These studies collectively explored various aspects of adverse birth outcomes and maternal morbidities among Afro-Latina and Afro-Caribbean populations. All studies except one were conducted in the U.S. and used retrospective data analysis of vital statistics. A summary of the study characteristics is available in Table 1.

### Low Birth Weight (LBW) and Small for Gestational Age (SGA)

The studies in our review suggest that Afro-Latinas exhibit a higher prevalence of low birth weight compared to their Non-Latina White and Black counterparts. Bediako (2015) highlighted significant disparities in low birth weight among various racial and ethnic groups. Specifically, there was a notable disparity in the prevalence of low birth weight in unadjusted models (7.18%, *p* < 0.001, 95% CI = 6.92–7.45) when compared to their non-Black Latina counterparts (5.80%, *p* < 0.001, 95% CI = 5.75–5.85) [25]. After adjusting for demographic factors, Afro-Latina mothers were observed to have a low birth weight prevalence of 5.11%, non-Afro-Latina (White Latinas) mothers had a prevalence of 4.24%, and non-Latina Black mothers had the highest prevalence at 7.38% [25]. Mydam’s 2019 study revealed that 7.9% of Afro-Latinas gave birth to low birth weight infants, higher than their White Latina counterparts (5.6%) [11].

Similar findings were supported among U.S.-born Afro-Latinas, who also had a higher prevalence of LBW and SGA compared to foreign-born Afro-Latinas. Within the Afro-Latina demographic, Puerto Rican (a U.S. territory) mothers showed the highest low birth weight prevalence (8.63%), and Mexican mothers had the lowest prevalence (6.39%) [25]. This highlights the complexity of factors contributing to disparities across the Latina demographic, where nationality may play a significant role [25]. Cuban mothers within the Afro-Latina group demonstrated the second-highest prevalence of low birth weight [25].

Further, nativity was an influential factor in low birth weight as US-born Afro-Latinas experienced a higher prevalence of low birth weight (8.9%) than their foreign-born Afro-Latina counterparts (6.1%) [11]. After adjusting for maternal age, education, marital status, parental acknowledgment, and WIC status, the odds ratios for foreign-born Afro-Latinas and non-Latina Whites became similar (OR = 1.03, 95% CI = 0.97–1.1) [11]. In contrast, US-born Afro-Latinas remained relatively high (OR = 1.47, 95% CI = 1.42–1.53) [11]. These similar odds ratios were sustained after adjusting for medical risk factors for low birth weight, including prenatal care initiation, pre-pregnancy BMI, parity, infant sex, and medical disease during pregnancy [11]. This finding aligned with that of Green et al., who found that Latina immigrants have children with higher birth weight infants (133 g heavier) compared to US-born Latinas. Among Afro-Latina women, having a Latina immigrant mother was associated with an approximately 40 g decrease in birth weight (*p* < 0.05) [5].

Infants of immigrant Afro-Latina mothers were, on average, 105 g heavier than infants of non-Latina Black mothers (*p* < 0.01) [5]. Notably, among U.S.-born non-Latina Black mothers and Afro-Latina first-time mothers, there was a significant difference in birth weight, with infants of Latina mothers outweighing those of non-Latina mothers by approximately 108 g (*p* < 0.01) [5]. Howard et al. also concluded that foreign-born Afro-Latinas had significantly lower risks of low birth weight than US-born Afro-Latina women [21].

On the other hand, Cocrot et al. findings indicated that among HIV-infected women within various ethnic groups, Afro-Latina foreign-born women had the lowest odds (AOR = 0.22, 95% CI = 0.07–0.71) of delivering an SGA infant than Non-Latina White foreign-born (AOR = 1.47, 95% CI = 0.53–4.11), non-Latina Black foreign-born (AOR = 0.72, 95% CI = 0.28–1.83), and White Latina foreign-born (AOR = 0.22, 95% CI = 0.15–0.61) women [19]. However, after adjusting for lifestyle behaviors, including smoking, alcohol, and illicit drug use during pregnancy, Afro-Latina foreign-born women (AOR = 0.60, 95% CI = 0.17–2.08) were observed to have higher odds of small for gestational age delivery than Latina White foreign-born women (AOR = 0.50, 95% CI = 0.23–1.09) [19].

### Preterm Birth

Findings suggested inconsistent disparities in preterm birth risks among U.S.-born Afro-Latina women compared to non-Latina White and Black (U.S.-born) women. Egbe et al. explored preterm birth risks across varying race, ethnicity, and nativity levels [26]. US-born Afro-Latina women had a significantly higher risk (AOR = 1.65, 95% CI = 1.23–2.23) of delivering moderately preterm infants than US-born White Latina women. U.S.-born Afro-Latina women also exhibited a higher relative risk of extreme, moderate, and late preterm births, although these findings were not statistically significant compared to [26]. Another study by Howard et al. found that among women of different ancestry groups (West Indian and Brazilian Black, African Black, European Black, Asian Black, Puerto Rican Black, Cuban Black, and South and Central American Black), Cuban Black women were the only group who had a higher risk of preterm birth and low birth weight compared to non-Latina Black (U.S.-born) women [21]. However, their study concluded that Afro-Latina mothers who are Puerto Rican, South, and Central American had relatively better preterm delivery outcomes [21].

### Maternal Morbidity (Gestational Diabetes-Related) Outcomes

Of the studies that met our inclusion criteria, only one article addressed maternal morbidity as an outcome of interest. The study conducted by Sinha et al. in 2003 explored the incidence of persistent postnatal glucose intolerance in a sample of Afro-Caribbean (including Afro-Latina population), Asian, and Caucasian women living in the United Kingdom [20]. The researchers observed that Afro-Caribbean women exhibited a significantly higher booking weight (i.e., initial weight recorded at the beginning of pregnancy), averaging 90.3 kg, compared to their Asian (*p* < 0.003) and Caucasian counterparts (*p* < 0.03) [20]. Similarly, the prevalence of a family history of diabetes was notably higher among Afro-Caribbean women (76%) than Asian (56%) or Caucasian women (31%) [20]. Despite the higher booking weight and more common family history of diabetes, Afro-Caribbean women have lower rates of postpartum impaired glucose tolerance/diabetes mellitus compared to Caucasian and Asian women [20]. When they were diagnosed with gestational diabetes, Afro-Caribbean women tended to be diagnosed with gestational diabetes earlier in their pregnancies (26.7 weeks) than Asian (28.4 weeks) and Caucasian (31.5 weeks) women [20]. These findings should be reviewed with an understanding that the sample of Afro-Caribbean in this study was small (*n* = 41, 19%) and that the term “Afro-Caribbean” encompasses individuals who may or may not identify with Latino ethnicity (e.g., participants from the Bahamas, Jamaica) [20]. Further, booking weight alone does not account for the participant’s height. Without that information, a BMI cannot be determined and may not necessarily mean the patient is overweight or obese.

## Discussion

The findings of this systematic review shed light on critical aspects related to maternal and infant disparities, particularly among the Afro-Latina population. Several key themes and trends have emerged as we have meticulously examined the available literature and synthesized the evidence. The available literature on Afro-Latina mothers and their infants is notably sparse, with only a limited number of studies addressing this specific Latina demographic globally. Although some research in our review has identified specific health disparities that Afro-Latina mothers and their infants face, mainly U.S born Afro-Latinas, such as a higher prevalence of low birth weight, small-for-gestational age, and preterm births compared to their ethnic counterparts [11, 19, 21, 25], our understanding of this complex phenomenon remains limited.

Overall, the homogenization of Latinos in research inherently omits key differences that may impact health outcomes [27]. The majority of the literature in this review that does account for Latina subgroups often dichotomizes between foreign-born and U.S.-born populations, which is an essential distinction with its own set of implications for maternal and child health outcomes, such as immigration status, country of origin, language barriers, and access to care. However, it is crucial to recognize that Afro-Latinas are present within both categories, rendering their position within the broader maternal and child health disparities landscape less defined.

Our systematic review revealed a notable trend. Compared to their foreign-born Afro-Latina peers, adverse birth outcomes were prevalent among U.S.-born Afro-Latinas. This phenomenon is well researched, as Latinos living in the U.S. longer or more acculturated Latinos have worse health outcomes than recent immigrants (e.g., Hispanic/Latino Paradox) [4]. This observation invites a critical examination of the underlying factors contributing to this disparity, potentially mirroring challenges faced by non-Latina Black perinatal women in the United States. Common threads, such as perceived discrimination within healthcare systems and limited access to prenatal resources, may be factors that may negatively affect both groups [6, 8]. The nuanced experiences and disparities Afro-Latina mothers face transcend the binary categorization of ethnicity. Therefore, a more comprehensive and inclusive approach is needed to address this population’s unique health challenges and needs effectively. To explore and address maternal morbidity adverse birth outcomes among Afro-Latina mothers and their infants, it is imperative to investigate these shared challenges to target areas of concern and develop potential interventions.

Further, this systematic review screened thousands of articles utilizing search terms related to maternal morbidity. A striking observation emerged: most of these studies primarily centered on adverse birth outcomes (e.g., infant birth weight). While the significance of studying birth outcomes cannot be overstated or challenged, it became evident that maternal morbidities, such as gestational diabetes and gestational hypertension, are critical factors that inherently influence both birth outcomes and infant health [28–30] had not received the attention it deserves among this particular population. Among the articles examined in this review, only one study in the UK specifically addressed maternal morbidity (gestational diabetes) as an outcome [20] in which Afro-Latinas did not have a significant difference in gestational diabetes compared to the other groups in the study, which signifies a critical research gap. We know maternal morbidity not only directly affects the well-being of mothers but also plays a pivotal role in shaping birth outcomes and the health of newborns [31]. Hence, we emphasize the pressing need for more research to prioritize maternal morbidities as outcomes of interest to understand, prevent, and mitigate adverse maternal disparities among this vulnerable population.

Afro-Latina is a valuable term in acknowledging the intersectional identity of this Latina subgroup. However, it has its limitations in research. Many studies rely on participants to self-identify their racial and ethnic identity. Afro-Latina self-identification can be influenced by various factors, including societal constructions, cultural backgrounds, personal experiences, stigmatization of Blackness, or ethnoracial dissonance [32, 33]; therefore, individuals of African descent within the Latina community may or may not use the term Afro-Latina to describe themselves [1, 8, 9, 34, 35]. Further, a limitation of this systematic review was the challenge of diversity in terminology. The complexity of Afro-Latina identity and its developmental process is not well understood by researchers [33]. Therefore, our team implemented a comprehensive and flexible search strategy, ensuring thorough screening regardless of the terminology used. However, search results can vary significantly depending on the terminology used in published studies. Some studies may use Afro-Latina, while others may use different terms (e.g., Black Latinas, Afro-Caribbean, Black Hispanic) or no specific terminology. This variability in terminology can lead to difficulties in identifying and aggregating relevant research on this particular population. This limitation highlights the importance of researchers remaining mindful of the evolving nature of racial and ethnic identities, such as Afro-Latinas when describing their study samples.

## Conclusions

Our systematic review aimed to evaluate the literature on maternal morbidity and adverse outcomes among Afro-Latinas and their infants. The studies examined have suggested the existence of maternal and infant health disparities among Afro-Latinas, particularly in the United States. Perinatal Afro-Latinas are at greater risk of experiencing preterm birth and having infants of low birth weight and small for their gestational age than White Latinas [18, 25]. Nevertheless, there remains a lack of research assessing racial inequities among Latinas in the context of maternal and child health and their related outcomes [6, 18]. Despite existing but scarce evidence, we know even less about drivers of racialized health inequity among Latinos [18]. Further, how these disparities may be influenced by social determinants of health, cultural considerations, and systematic and implicit biases within the healthcare system are needed for a more comprehensive and nuanced understanding of Afro-Latina maternal and infant health.

Despite the growing recognition of racial and ethnic diversity among the Latino population in the United States, Afro-Latinas’ health has been inadequately studied due to arbitrary categorization of race and ethnicity among Latino/a’s in public health and health equity research [18]. With a significant focus on improving outcomes among perinatal Black women, exploring the unique challenges and disparities faced by perinatal Afro-Latinas should be a public health priority. By doing so, we can work towards equitable maternal and infant health outcomes, ensuring that Afro-Latinas receive the necessary care and support they deserve during this critical stage of life.

## Figures and Tables

**Table 1:** Details of included studies

Author, year, country	Study design	Study sample	Afro-Latina sample description in article	Outcome variables	Key findings
Egbe 2022 United States	Cross-sectional	*N* = 12,107 Total: *N* = 499,259	Hispanic Black*	*BO:* preterm birth	All ethnic/native subgroups of Black women (including Afro-Latinas) had a significantly increased risk of extreme preterm birth compared to US-born non-Latina White women
Mydam 2019 United States	Cross-sectional	*N* = 52,361 Total: *N* = 7,865,264	Black Latina*	*BO:* infant weight	The low birth weight (LBW) rate among Black Latinas (7.9%) was higher than that among White Latinas (5.6%), and it varied based on nativity: US-born Black Latinas had a higher LBW rate (8.9%) compared to foreign-born Black Latinas (6.1%) US-born Black Latinas had the second-highest LBW rate (8.9%) following non-Latina Blacks (11.0%) After adjusting for sociodemographic factors (model 2), the odds ratios (ORs) for foreign-born Black Latinas became nearly identical to non-Latina Whites, while US-born Black Latinas still had 47% higher odds of LBW
Bediako 2015 United States	Cross-sectional	*N* = 37,398 Total: *N* = 2,970,315	Black Hispanic*	*BO:* preterm birth Birth weight Small-for-gestational age	Non-Latina Black mothers had the highest rates of adverse birth outcomes, including low birth weight (LBW), preterm birth (PTB), and small for gestational age (SGA). White Latina mothers had birth outcomes comparable to non-Latina White mothers Afro-Latina mothers had intermediate rates of adverse birth outcomes, with rates closer to White Latina mothers than non-Latina Black mothers
Green 2014 United States	Cross-sectional	*N* = 9143 Total: *N* = 9143	Black Hispanic*	*BO:* infant weight	Latina Black mothers give birth to infants with higher birth weights infants compared to non-Latina Black mothers
Howard 2006 United States	Cross-sectional	*N* = 19,373 Total: *N* = 1,213,272	South and Central American Black* Puerto Rican Black* Cuban Black*	*BO:* preterm birth Low birth weight	Foreign-born Black women had lower risks of preterm birth and low birth weight than American born Black women, with the exception of Cubans Among South/Central American and Cuban women, maternal ancestry and nativity was more predictive of low birth weight than preterm birth
Sinha 2003 UK	Cross-sectional	*N* = 41 Total: *N* = 221	Afro-Caribbean	*MM:* gestational diabetes Impaired glucose tolerance (IGT)	The booking weight was significantly greater in Afro-Caribbean women (90.3 kg) compared with both Asian (*P* < 0.003) and Caucasian women (*P* < 0.03) GDM in the index pregnancy was detected in the Afro-Caribbean women at a mean of 26.7 weeks compared with 28.4 weeks in Asian women and 31.5 weeks in the Caucasian cohort
Cocroft 2002 United States	Cross-sectional	*N* = 186 Total: *N* = 2525	Black Latina*	*BO:* Small-for-Gestational Age	SGA for Afro-Latinas (foreign and us born combined) *n* = 33.9% compared to non-Latina white women *n* = 12.7% US-born (including Puerto Rican–born) White Latina and Afro-Latina mothers had higher proportions of SGA babies than their respective foreign-born counterparts

Outcome categories:

* Self-reported race by participants; *MM*, maternal morbidity; *BO*, birth outcomes

## Data Availability

Availability of review materials are available upon request.
